# Toll-Like Receptor 4 Activation Promotes Multiple Myeloma Cell Growth and Survival Via Suppression of The Endoplasmic Reticulum Stress Factor Chop

**DOI:** 10.1038/s41598-019-39672-7

**Published:** 2019-03-01

**Authors:** Tina Bagratuni, Aimilia D. Sklirou, Efstathios Kastritis, Christine Ivy Liacos, Christina Spilioti, Evangelos Eleutherakis-Papaiakovou, Nikolaos Kanellias, Maria Gavriatopoulou, Evangelos Terpos, Ioannis P. Trougakos, Meletios A. Dimopoulos

**Affiliations:** 1 Department of Clinical Therapeutics, National and Kapodistrian University of Athens, Athens, Greece; 2 Department of Cell Biology and Biophysics, Faculty of Biology, National and Kapodistrian University of Athens, Athens, 15784 Greece

## Abstract

Despite recent biomedical improvements in treating Multiple Myeloma (MM), the disease still remains incurable. Toll like receptors (TLRs) provide a link between innate and adaptive immune responses and hence potentially correlate inflammation to cancer. Although the regulatory role of TLRs in MM has been under investigation the underlying mechanisms remain unclear. In this study we assayed the function of TLR4 in MM cell lines and in MM patients’ samples. We found that lipopolysaccharide-mediated TLR4 activation increased MM cells proliferation and decreased endoplasmic reticulum (ER) stress-induced apoptosis. Furthermore, we observed that either the endogenous CHOP expression or the ER stress-mediated CHOP induction, were suppressed by TLR4 activation or its overexpression in MM cell lines; TLR4 induction also suppressed ER stress-induced apoptotic signals. In support, TLR4 gene expression silencing in MM cell lines significantly decreased cell proliferation and promoted CHOP and ATF4 upregulation. TLR4 activation was also able to partially abrogate the effect of bortezomib in MM cell lines by suppressing PERK, ATF4 and phospho-eIF2A. We suggest that TLR4-mediated disruption of ER stress responses contributes to MM cells proliferation and suppresses ER-dependent death signals.

## Introduction

Survival and proliferation of multiple myeloma (MM) plasma cells largely depends on the bone marrow microenvironment and the presence of local and circulating cytokines. Cytokines such as interleukin (IL)-6 and tumor necrosis factor α (TNF-α)^1–3^ seem to play a critical role on MM cell survival. Although several studies have proposed a potential role of inflammatory or infectious responses to MM pathogenesis and/or progression^4–6^, the underlying molecular mechanisms remain elusive. The Toll-like receptor (TLR) family of receptors is activated during infection in order to signal to immune cells the presence of invading pathogens and to regulate the growth of human B lymphocytes^7^. The activation of the TLR signaling pathway switches on transcriptional programs that coordinate adaptive responses to specific insults. Reportedly, TLR activation may also be implicated in B-cell related malignancies including MM as it was found that the transcript levels of *TLR4* and *TLR9* were higher in bone marrow mononuclear cells (BMMCs) from MM patients as compared to those from healthy donors^7^. It was also showed that the ligands of TLR4 and TLR9, i.e. lipopolysaccharide (LPS) and CpG oligonucleotides respectively, promoted the growth of MM cells which could be attenuated by blocking NF-κB and IL-6 activities^7^; however, there has been no mechanistic explanation for this effect in MM cells.

In case of accumulation of misfolded and/or unfolded proteins in the endoplasmic reticulum (ER) the unfolded protein response (UPR) is activated which aims to restore normal cell function by maintaining the balance of protein production and protein folding. Activation of the UPR results in increased production of molecular chaperones that are involved in protein folding, such as GRP94 and CHOP (CCAAT/-enhancer-binding protein homologous protein); these chaperones are also involved in transmitting pro-death signals in conditions of intense DNA damage or ER stress^8^. Upon increased ER stress, the ATF6 and PERK/eIF2A are activated leading to the induction of ATF4 translation and to CHOP upregulation^9–11^. Therefore, the presence of correctly folded proteins (e.g. nascent IgGs) within the ER provides an effective checkpoint of cell survival and consequently plasma cell development.

Previous studies have revealed that prolonged ER stress occurs in response to microbial infections, particularly in cells exposed to LPS, a major activator of TLR4. A possible link between the ATF4-CHOP branch and TLR signaling has been reported, where pre-treatment with LPS in mice subjected to ER stress demonstrated an inhibitory effect in CHOP expression and apoptosis in splenic macrophages, renal tubule cells, and hepatocytes^12,13^. Similarly, it was found that TLR4 and TLR2 specifically activated the IRE1a arm of the UPR and its downstream target XBP1, a pro-survival transcription factor that is essential for plasma cells differentiation^14^. These findings suggested that activation of IRE1a acted in synergy with TLR activation for cytokine production, indicating a possible link of TLR4 signaling and of the UPR pathway in cell survival and proliferation; it is expected that this effect is likely maximized in cells (e.g. MM cells) that are highly dependent for their survival on the effective action of the UPR pathway. In support, a recent study by our group has shown that certain polymorphisms in TLR4 pathway are associated with poor outcome in myeloma patients^15^.

We report herein the role of TLR4 signaling on MM cells proliferation and survival, which may also relate to acquired resistance of MM cells to therapeutic proteasome inhibitors.

## Results

### Human Myeloma cells express TLR4

**Figure 1:**
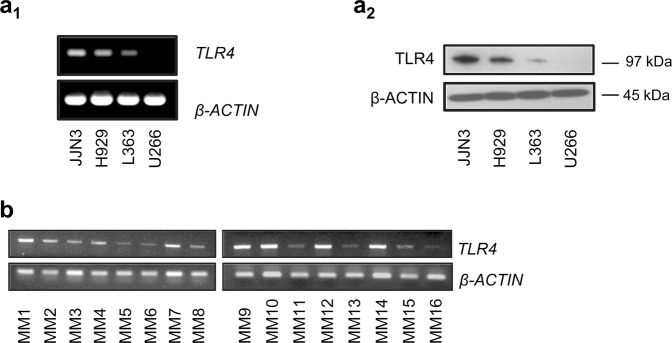
*TLR4* mRNA (**a**_**1**_) and protein (**a**_**2**_) expression in MM cell lines. (**b**) *TLR4* mRNA expression of 16 CD138^+^ selected MM patients as determined by PCR and agarose gel electrophoresis. Probing with β-ACTIN was used as total protein loading reference, whereas *β-ACTIN* gene expression was used as reference for RNA input. β-ACTIN probing and *β-ACTIN* mRNA expression were used as reference for total protein and mRNA input, respectively.

We first screened 4 MM cell lines (L363, H929, U266 and JJN3 and RPMI-LR5) for the TLR4 mRNA (Fig. 1a1, Suppl. Fig. S1) and protein (Fig. 1a_2_, Suppl. Fig. S2) expression. TLR4 was differentially expressed among the above MM cell lines with JJN3 and H929 having the highest and L363 and U266 the lowest expression levels. A highly positive correlation is shown between TLR4 mRNA and protein expression (r = 0.99).

The expression of *TLR4* mRNA was also determined in CD138^+^ myeloma cells isolated from the bone marrow of 16 patients at time of diagnosis. Gene expression analyses showed that most CD138^+^ myeloma cells express high levels of *TLR4* (Fig. 1b, Suppl. Fig. S3). We also examined public depositories of Gene Expression Profile (GEP) data and found that indeed TLR4 is expressed in CD138^+^ selected cells (GSE68891, GDS1284) (not shown).

Thus, the TLR4 signaling pathway likely plays a central role in plasma cell biology.

### TLR4 activation increases MM cells viability and proliferation

**Figure 2:**
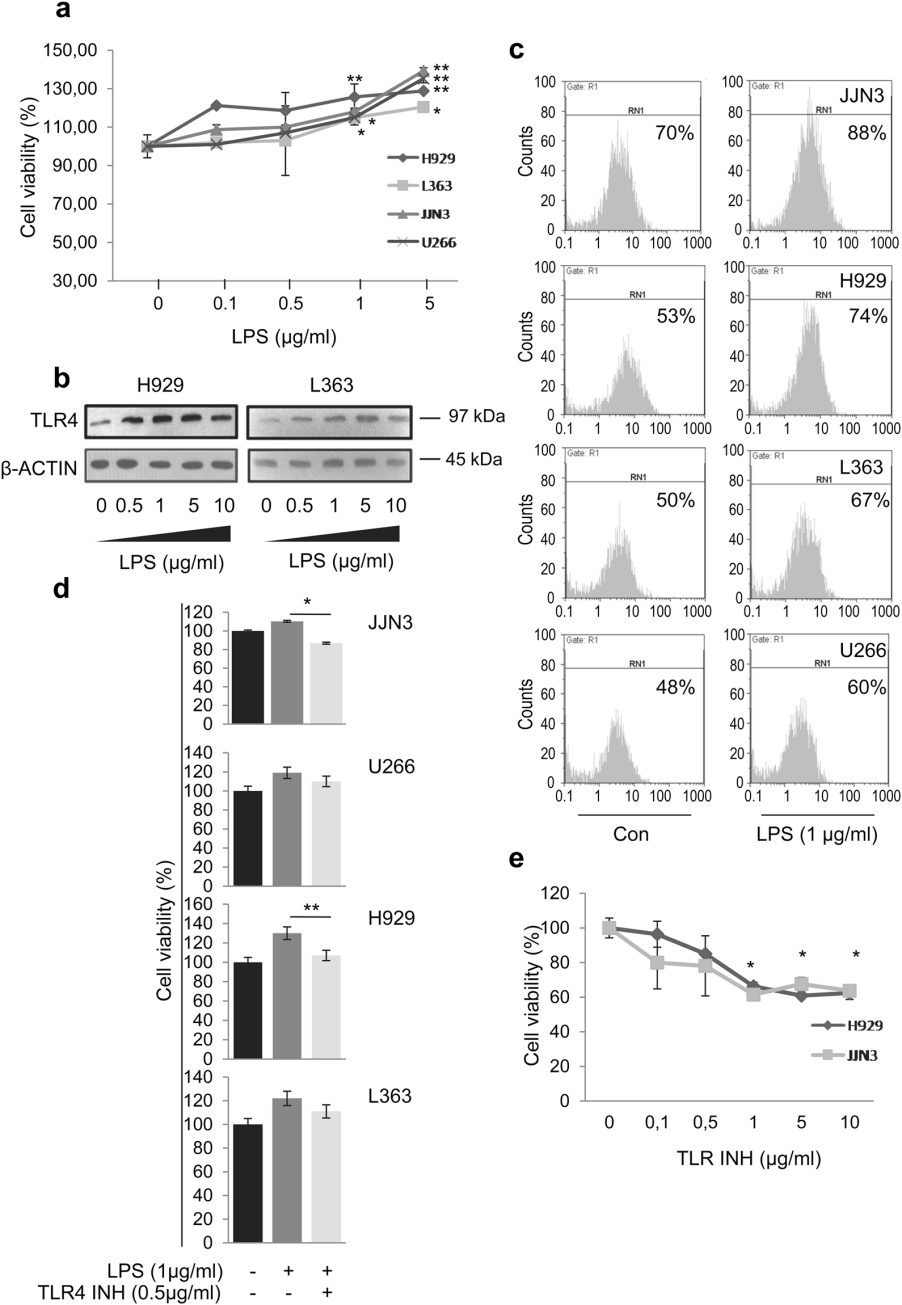
(**a**) Cell viability of MM cells exposed to increasing doses of LPS for 24 h. (**b**) Immunoblotting analysis of TLR4 protein expression levels in H929 and L363 cell lines after incubation with various LPS concentrations. Probing with β-ACTIN was used as total protein loading reference. (**c**) Flow cytometry analysis for TLR4 expression of MM cell lines before and after exposure to 1 μg/ml LPS for 24 h. (**d**) Cell viability of MM cell lines pre-treated with 0.5 μg/ml TLR4 inhibitor for 24 h before stimulation with LPS for 24 h. (**e**) Cell vViability of H929 and JJN3 cell lines exposed to increasing concentrations of TLR4 inhibitor for 48 h. β-ACTIN probing was used as reference for total protein input.

We then investigated the effect of LPS-mediated TLR4 activation on MM cells viability. Specifically, we studied the effects of various concentrations of LPS (0.1, 0.5, 1 and 5 μg/ml) on MM cells viability using the WST1 viability assay. LPS significantly enhanced MM cells viability (as compared to non treated control cells) in all cell lines after 24 h. As shown in Fig. 2a, treatment with 1 μg/ml LPS significantly increased viability of H929, JJN3 and U266 cells by around 25%, 20% and 15% respectively while for L363 cells viability was found to increase was increased (*vs*. non treated cells) by 10% (p = NS). In support, cell exposure to higher doses (5 μg/ml) of LPS significantly increased MM cell viability (*vs*. non treated cells) by almost 40% and 35% for JJN3 and U266 cells, respectively and by 28% and 20% for H929 and L363cells, respectively. In addition we studied the effects of different LPS concentrations and at various time points (24, 48 and 72 hours), on the proliferation of the MM cell lines using the BrdU proliferation assay. Our results indicate that the most prominent effects of LPS on MM cell lines are seen at 24 hours of treatment (Suppl. Fig. S4). Thus LPS-mediated TLR4 activation affects dose-dependently even cell lines which express low TLR4 levels.

To examine whether LPS stimulation affects TLR4 expression, we also evaluated TLR4 protein expression levels upon LPS stimulation at various concentrations (0.1, 0.5, 1, 5 and 10 μg/ml). We observed that the TLR4 expression levels increase dose-dependently following treatment with increasing LPS doses (Fig. 2b, Suppl. Fig. S5). A systemic correlation analysis between the expression of TLR4 and viability in H929 (r = 0.59) and L363 (r = 0.54) cells, showed the positive correlation and linkage between TLR4 expression and viability. The effects of LPS on TLR4 expression were also evaluated by flow cytometry, where we found that treatment of JJN3, H929, L363 and U266 cells with LPS for 24 h increased TLR4 surface expression by 18%, 21%, 17% and 12%, respectively (Fig. 2c).

To determine whether the increased MM cells proliferation after LPS treatment was due to LPS-mediated TLR4 activation, cells were pre-treated with a TLR4 inhibitor (TAK-242) for 24 h before stimulation with LPS for another 24 h; this inhibitor acts by blocking the intracellular signaling domain of TLR4 and potently suppresses ligand-dependent and -independent TLR4 signaling. We found that pre-treatment of JJN3 and H929 cells with the TLR4 inhibitor (0.5 μg/ml) significantly abrogated the viability effects of LPS (1 μg/ml) by ~40%, and 25%, respectively while in U266 and L363 cells that express low endogenous levels of TLR4 levels, the reduction was less pronounced ~10%, (p = NS) (Fig. 2d). Notably, treatment of MM cell lines with medium or high TLR4 levels, (H929 and JJN3, respectively) with the TLR4 inhibitor alone (0.1, 0.5, 1, 5 and 10 μg/ml concentrations), significantly inhibited their viability dose-dependently by up to 40% (Fig. 2e) indicating that at least with medium/high TLR4 expression levels basal TLR4 signaling contributes to MM cell viability in the absence of LPS induction.

**Figure 3:**
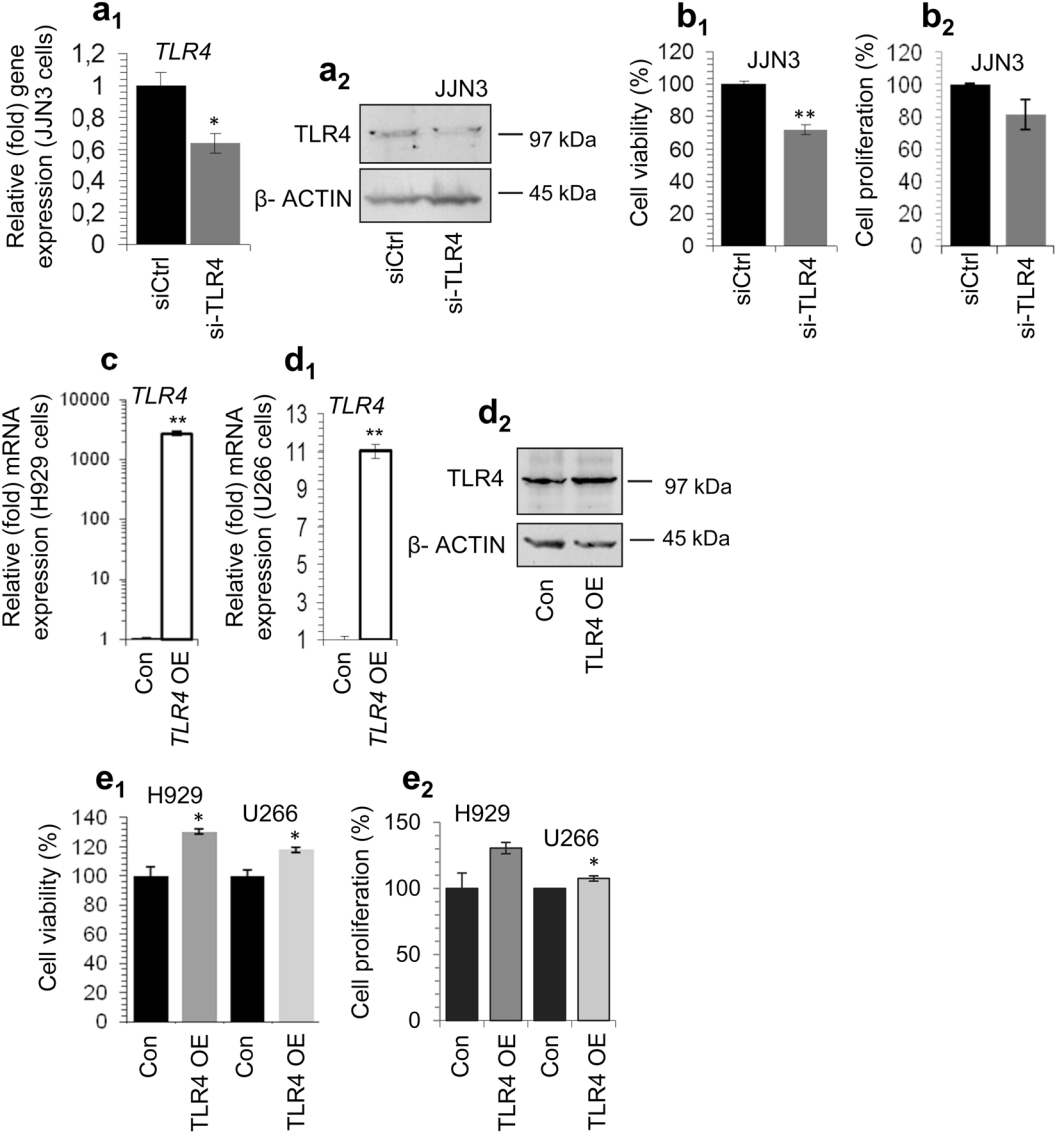
(**a**_1_) Q-PCR assay of *TLR4* mRNA expression levels after transfecting JJN3 cell line with *TLR4* RNAi oligonucleotides or a non-targeting pool (siCtrl) for 48 h. (**a**_**2**_) Representative immunoblotting analyses of protein samples probed with an antibody against TLR4 after *TLR4* RNAi for 48 h. (**b**_**1**_) % cell viability and (**b**_**2**_) % proliferation of JJN3 cells after *TLR4* RNAi for 48 h. (**c**,**d**_**1**_) Q-PCR expression analyses of *TLR4* mRNA expression levels after transfecting H929 (**c**) and U266 (**d**_**1**_) cell lines with the pCMV6-TLR4 construct or a pCMV6 empty vector for 48 h. (**d**_**2**_) Immunobloting analyses of U266 cells transfected with pCMV6-TLR4 or pCMV6 vector for 48 h; protein samples were probed with an antibody against TLR4. (**e**_**1**_) % cell viability and (**e**_**2**_) % proliferation of H929 and U266 cells after transfection with the pCMV6-TLR4 construct or with the empty pCMV6 vector for 48 h. β-ACTIN probing and *β-ACTIN* mRNA expression were used as reference for total protein and mRNA input, respectively. Cells transfected with the pCMV6-TLR4 construct are labelled as TLR4 OE (TLR4 overexpression), whereas cells transfected with the pCMV6 empty vector are labelled as Con (control).

We then asked whether RNAi-mediated silencing of *TLR4* gene expression could phenocopy the TLR4 inhibitor-mediated effects (see Fig. 1a); these assays were done in JJN3 cells that express high endogenous TLR4 levels and are responsive to LPS. Despite the well established differences between the siRNA- (where it is expected that in spite of TLR4 downregulation cells will retain residual TLR4 activity) and inhibitor- (likely the most of the signaling is quenched) mediated effects, we observed that effective knock down of the *TLR4* mRNA (Fig. 3a_1_) and protein (Fig. 3a_2_, Suppl. Fig. S6) expression decreased JJN3 cells viability (Fig. 3b_1_) and tended to reduce their proliferation rate (Fig. 3b_2_).

On a complementary approach, by using a pCMV6-TLR4 construct we overexpressed TLR4 in H929 (Fig. 3c) and U266 (Fig. 3d_1_) cells, as these MM cell lines express medium or low (compared to JJN3 cells) TLR4 levels; notably, in spite of very high *TLR4* mRNA expression levels in transfected cells (Fig. 3c,d_1_) the TLR4 protein was only mildly overexpressed (Fig. 3d_2_, Suppl. Fig. S6) indicating the existence of post-transcription mechanisms that suppress high TLR4 protein expression levels. Yet, even mild TLR4 protein induction reduced spontaneous cell death of both the H929 and U266 cells (Fig. 3e_1_) and increased cell proliferation rates (Fig. 3e_2_); the use of another pUNO1-TLR4 (InvivoGen) construct yielded similar results (not shown).

Thus, TLR4 signaling in MM cells transmits basal and LPS-mediated pro-survival and proliferation signals.

### Pre-treatment of cells with LPS leads to marked suppression of CHOP expression coupled with anti-apoptotic effects in MM cells

**Figure 4:**
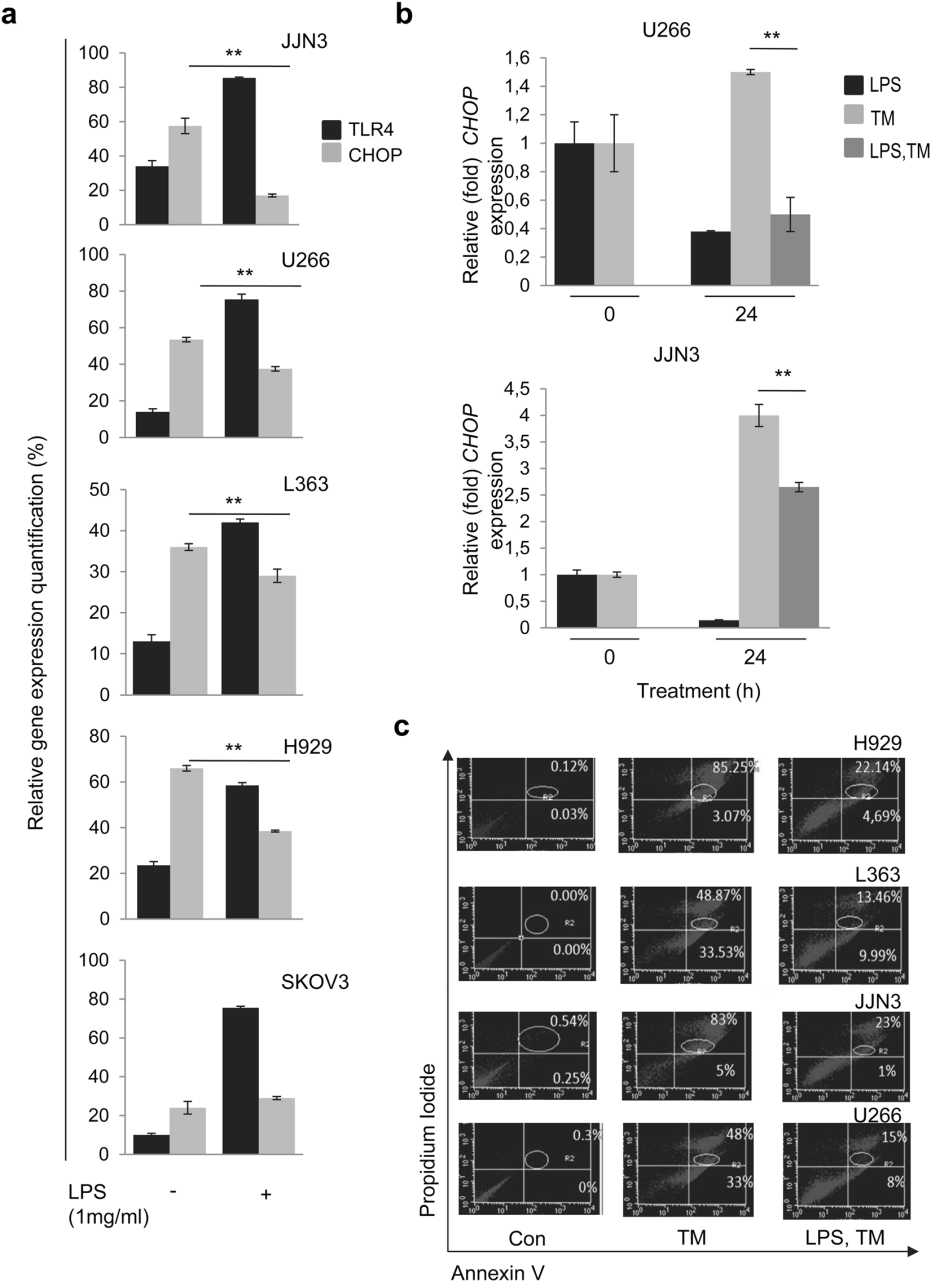
(**a**) Q-PCR expression analyses of *TLR4* and *CHOP* mRNA expression levels in MM cell lines JJN3, U266, L363 and H929 as well as in ovarian cancer cell line SKOV3 after incubation with LPS 1 μg/ml for 24 h. (**b**) Q-PCR expression analyses of *CHOP* mRNA expression levels of JJN3 cells pre-treated with LPS (1 μg/ml) for 2 h and then subjected to ER stressor TM. (**c**) Annexin-PI staining of MM cell lines subjected to TM for 24 h with or without LPS pre-treatment for 2 h; to set up compensation and quadrants, unstained cells, cells stained only with Annexin and cells stained only with PI were used as controls. *β-ACTIN* gene expression was used as reference for RNA input.

It has been shown that under prolonged ER stress, immune cells such as macrophages carry out essential protein synthesis and in parallel suppress CHOP-induced apoptosis in a TLR-dependent pathway; while still benefiting from the other cytoprotective arms of the UPR^12^. CHOP levels are relatively low under normal conditions in MM and are markedly induced by a variety of stressful conditions, including nutrient deprivation and/or treatment of cells with certain toxins^16,17^. Accumulation of misfolded proteins is a strong signal of integrated stress response and UPR activation, that among others results in CHOP activation (a marker of ER-stress-induced apoptosis) and of growth arrest DNA damage 34 (GADD34)^11,18,19^. As myeloma plasma cells produce large amount of immunoglobulins, integrated stress response and UPR is constantly activated and CHOP expression levels are higher than in normal plasma cells. Thus, the role of PERK-CHOP signaling is central to UPR-induced apoptosis, indicating that drug resistance in MM cells may relate to suppression of this apoptotic axis. Since a link between the ATF4-CHOP branch and TLR4 signaling has been reported^12,13^, we sought to determine whether TLR4 signaling modulates endogenous CHOP expression in MM cells. Thus, we treated MM cell lines with LPS (1 μg/ml) for 24 h. We found that TLR4 activation by LPS significantly decreased CHOP transcript levels in all four multiple myeloma cell lines studied (Fig. 4a). A systemic correlation analysis shows a highly negative correlation between the two genes (r = −0.97).

We further tested the specificity of these effects in a non-B cell line, namely SKOV3 ovarian cancer cells. Interestingly, although LPS pre-treatment increased *TLR4* mRNA expression in SKOV3 cells, *CHOP* mRNA levels were not altered before or after treatment with LPS (Fig. 4a). These results indicate that likely the TLR4-CHOP regulator axis affects cells which are highly dependent (e.g. MM cells) to the integrated stress response.

Next, in order to determine whether LPS could suppress CHOP expression even under conditions of sustained ER stress, U266 and JJN3 cells were treated with 1 μg/ml LPS for 2 h and were then subjected to ER stress conditions by treatment with Tunicamycin (TM) for 24 h; these conditions are known to upregulate CHOP in MM cells^8,20^. We found that while TM increased *CHOP* mRNA expression levels by ~50% at 24 h, this effect was significantly abolished in both cell lines upon pre-treatment with LPS for 24 h (Fig. 4b). Moreover, we investigated the effect of LPS on TM-mediated apoptosis. MM cells were pre-treated with LPS and TM and were then subjected to Annexin-PI staining to determine apoptotic events. TM-treated JJN3, H929, L363 and U266 cells showed an enhancement (as compared to non treated cells) of Annexin^+^/PI^+^ stained cells after 24 h of treatment by TM; also, in support to our previous findings, LPS addition inhibited (as compared to only TM-treated cells) apoptosis in JJN3, H929, L363 and U266 cells (Fig. 4c). Thus, even during sustained ER stress (e.g. induced by a selective ER stressor) TLR4 activation exerts anti-apoptotic effect on MM cells by inhibiting the CHOP pathway.

**Figure 5:**
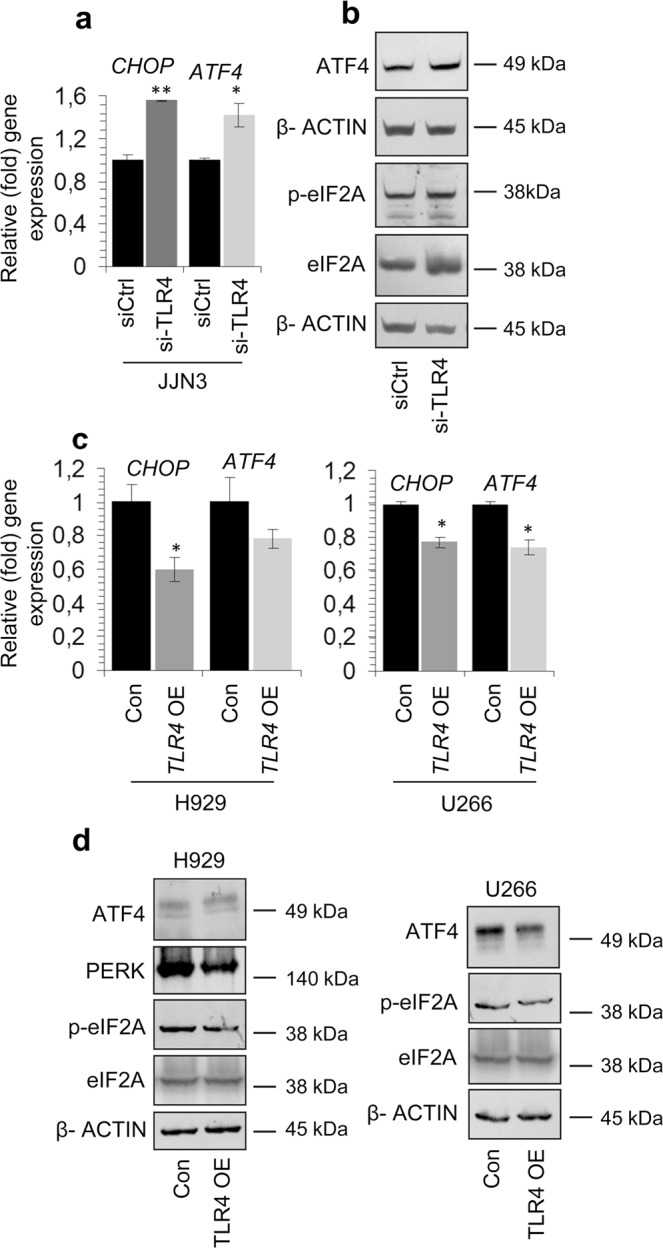
(**a**) Q-PCR expression analyses of *CHOP* and *ATF4* mRNA expression levels after transfecting JJN3 cells with *TLR4* RNAi oligonucleotides or a non-targeting pool (siCtrl) for 48 h. (**b**) Representative immunoblotting analyses of protein samples (after *TLR4* RNAi for 48 h) probed with antibodies against ATF4, p-eIF2A and eIF2A. (**c**) Q-PCR assay of *CHOP* and *ATF4* gene expression levels in H929 and U266 cell lines following transfection with the pCMV6-TLR4 construct or the pCMV6 empty vector for 48 h. (**d**) Immunoblotting analyses of ATF4, PERK, p-eIF2A and eIF2A after transfection of H929 and U266 cells with pCMV6-TLR4 or pCMV6 vector for 48 h. Probing with β-ACTIN was used as total protein loading reference, whereas *β-ACTIN* gene expression was used as reference for RNA input. TLR4 OE denotes cells transfected with the pCMV6-TLR4 construct and Con cells transfected with the pCMV6 empty vector.

Consistently, TLR4 knock down in JJN3 cells promoted the induction of *CHOP* and *ATF4* genes (Fig. 5a), as well as of ATF4 protein expression levels; it also increased eIF2A phosphorylation (Fig. 5b, Suppl. Fig. S7). On the contrary, mild TLR4 overexpression in H929 or U266 cells for 48 h decreased *CHOP* and *ATF4* genes expression levels (Fig. 5c); it also reduced PERK protein expression levels and eIF2A phosphorylation in H929 cells and ATF4 protein expression levels and eIF2A phosphorylation in U266 cells (Fig. 5d, Suppl. Fig. S7). Thus, TLR4 is a negative regulator of integrated stress response (e.g. *CHOP*, *ATF4*) genes.

### TLR4 signaling abrogates BTZ effect in MM cells

**Figure 6:**
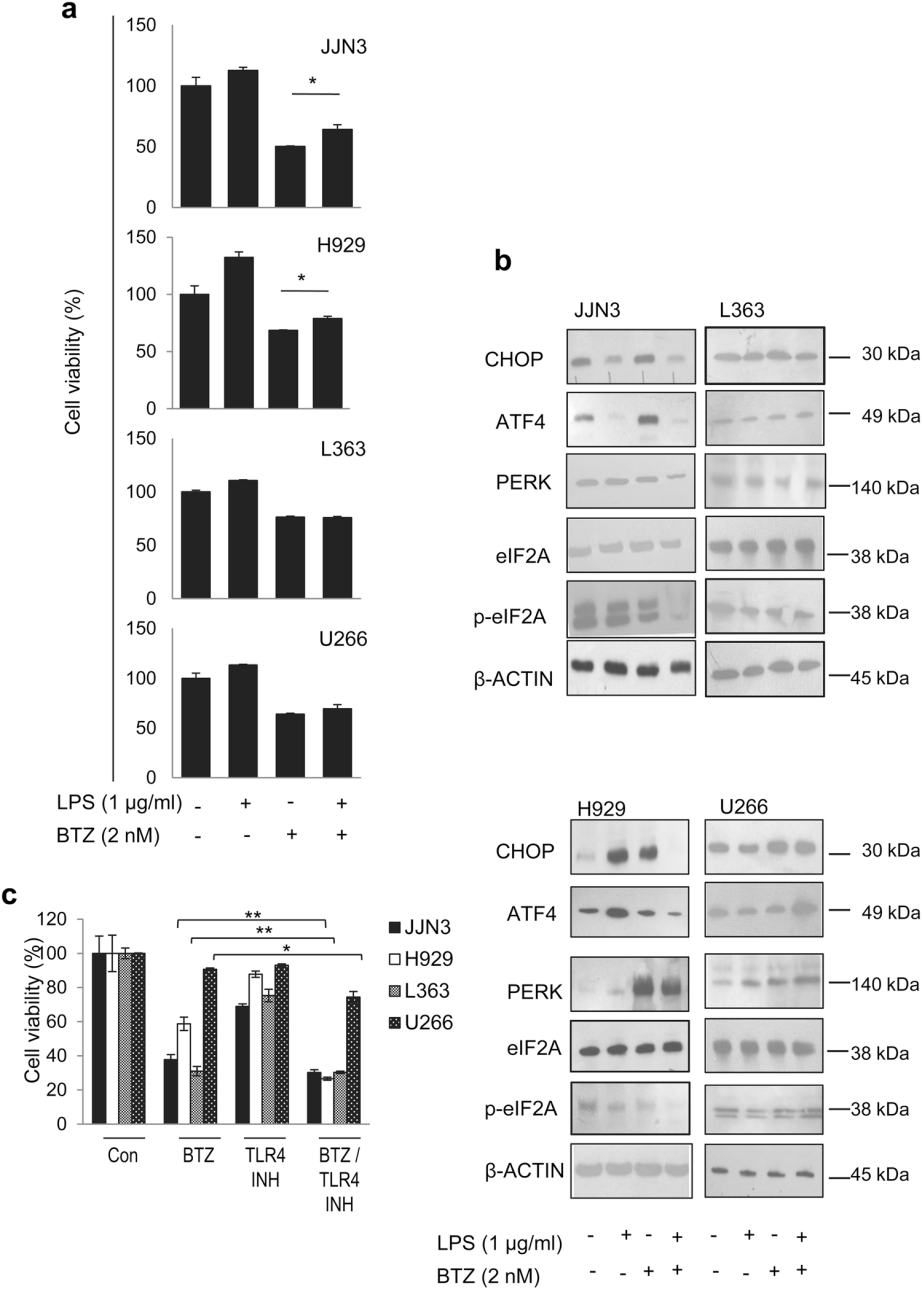
(**a**) Cell viability of cells pre-treated with LPS (1 μg/ml) for 2 h and then exposed to 2 nM BTZ for 24 h. (**b**) Representative immunoblotting analyses of protein samples probed with antibodies against CHOP, ATF4, PERK, eIF2Α and p-eIF2Α in MM cell lines pre-treated with 1 μg/ml LPS and then incubated with BTZ (2 nM) for 24 h. (**c**) Cell viability of MM cell lines pre-treated with TLR4 inhibitor (0.5 μM) for 48 h and then exposed to BTZ (2 nM) for 24 h. Probing with β-ACTIN was used as total protein loading reference.

Proteasome inhibitors and specifically BTZ, a therapeutic proteasome inhibitor, induce ER stress and upregulate the expression of UPR modules including *BiP* and *CHOP* at both mRNA and protein levels^21^. Given the usage of proteasome inhibitors (e.g. BTZ) in the treatment of MM, we then evaluated the effects of TLR4 signaling in MM cells after BTZ treatment. MM cells were pre-treated with 1 μg/ml LPS for 2 h and were then subjected to 2 nM of BTZ for 24 h. Pre-treatment with LPS partially abrogated the efficacy of BTZ in H929 and JJN3 cells, as it significantly increased cell viability (Fig. 6a); on the contrary, no significant effects were seen in L363 and U266 cells which express low TLR4 levels. Immunoblotting analyses revealed that LPS stimulation decreased protein levels of both CHOP and ATF4 in JJN3 cells which express high TLR4 levels (Fig. 6b, Suppl. Fig. S8). Notably, although CHOP transcript levels were reduced upon LPS stimulation in all cell lines (Fig. 4a), the reduction of CHOP transcript levels in H929, L363 and U266 cells was not accompanied with reduced expression of the CHOP and ATF4 proteins (Fig. 6b, Suppl. Fig. S8). This readout indicates that these less responsive to TLR4 cells, are likely less dependent on TLR4 signaling for their survival and hence they evade apoptosis via other pathways. Since TLR4 signaling is involved in additional UPR pathways^14,22,23^, we also investigated the activation of the other branches of UPR, namely ATF6 and XBP1 splicing which mainly deliver pro-survival signals^8,24,25^. We found that LPS pre-treatment did not affect ATF6 protein expression or XBP1 splicing as compared to non treated cells (Supp. Fig. S9). Thus, LPS-mediated activation of TLR4 suppresses CHOP expression in TLR4-responsive cells without affecting the other two UPR branches, indicating that TLR4 signaling is exclusively linked to the CHOP-ATF4 pathway. Furthermore, LPS pro-survival effect in BTZ-treated JJN3 and H929 cells was accompanied with CHOP downregulation (Fig. 6b, Suppl. Fig. S8). LPS pre-treatment reduced (as compared to only BTZ treated cells) PERK, ATF4 and p-eIF2A protein expression levels in BTZ treated JJN3 and H929 cells (Fig. 6b, Suppl. Fig. S8); these effects were less pronounced in L363 and U266 cells suggesting that the anti-apoptotic effects of LPS likely correlate with MM cells addiction to increased TLR4 signaling. Finally, we tested the activity of BTZ in combination with TLR4 inhibitor in all four MM cell lines. Cells were pre-treated with 0.5 μM of the TLR4 inhibitor (see above; IC_80_ dose) for 48 h and then BTZ (2 nM) was added for 24 h. We found that pre-treatment of JJN3, H929 and U266 cells with the TLR4 inhibitor significantly enhanced the anti-viability effect of BTZ when compared to solely BTZ treated cells (Fig. 6c). Therefore, TLR4 stimulation reduces the effect of BTZ on MM cells, while TLR4 inhibition increases the BTZ anti-proliferative effects.

Overall, TLR4 stimulation reduces the apoptotic effect of BTZ by modulating the integrated stress response components representing thus a potential mechanism of drug resistance.

### TLR4 signaling is activated in MM patients treated with therapeutic BTZ

**Figure 7:**
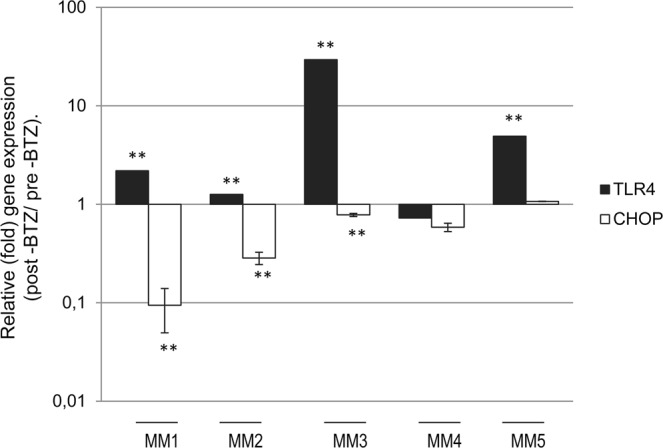
Q-PCR expression analyses of *TLR4* and *CHOP* gene expression levels in MM patient samples before and after BTZ treatment. *β-ACTIN* gene expression was used as reference for total RNA input.

Given our findings in MM cells, we then examined the impact of therapy with BTZ on *TLR4* and *CHOP* mRNA expression in primary plasma cells isolated from MM patients. To this end, CD138^+^ plasma cells were selected from MM patient samples before and at day 7 post BTZ-based therapy. We found that *TLR4* gene expression was significantly up-regulated (as compared to pre-treatment state) in most patients on day 7 post-BTZ therapy; this effect was accompanied (in most patients) with a tendency for lower expression levels of *CHOP* mRNA (Fig. 7).

Thus, TLR4 signaling may provide an additional mechanism of resistance to therapeutic proteasome inhibitors.

## Discussion

Although previous studies have implicated TLR4 signaling in MM cells survival^26–32^, our study investigated the role of TLR4 in malignant plasma cells survival through its potential interaction with the integrated stress response components. Our results provide evidence that LPS-mediated TLR4 stimulation promotes myeloma cell growth and survival by suppressing apoptosis associated with integrated stress response and UPR activation through PERK-CHOP signaling. A schematic representation demonstrating the possible interaction of TLR4 activation with integrated stress responses is shown in Suppl. Fig. S10.

The expression pattern of *TLR4* has been previously investigated in myeloma, and it was found to be highly expressed in most myeloma cell lines, as well as in primary myeloma cells^29–31,33^. Our study confirms that TLR4 is expressed in myeloma cells as shown by the expression pattern of most myeloma cell lines examined and of 16 CD138^+^ selected myeloma patient bone marrow samples. Consistent with the mRNA and protein TLR4 expression levels, flow cytometry analysis revealed that TLR4 protein was present on the surface of MM cell lines. L363 and U266 cells expressed the lowest levels of TLR4, which is also supported by other studies^29,31^; yet, even in these cells activation of TLR4 signaling via LPS stimulation promoted MM cell viability and proliferation.

In addition to high TLR4 expression in MM cells, previous studies have also shown that TLR4activation in plasma cells results in massive IgG production and secretion^33^. We hypothesized that the major role of TLRs in both innate immunity and as initiators of adaptive immunity is likely based on the fact that this pathway provides a protective effect in the cells of the immune system under conditions of prolonged ER stress driven by the inflammatory reaction and/or toxic products of the invading organisms. Thus, since MM cells rely heavily on the UPR pathway for their survival^21,34–37^, a link among UPR and integrated stress response and TLR4 activation may exist. To untangle this link we focused on the status ER stress response in regards to TLR4 activation by LPS treatment. We found that TLR4 activation by LPS stimulation increased MM cell viability and exerted anti-apoptotic effects. This effect was accompanied by the suppression of ATF4-CHOP pathway in all cells assayed at the transcript level and was particularly enhanced seen in JJN3 cells where CHOP and ATF4 levels were also downregulated at protein level. This output likely relates to the fact that JJN3 cells express high TLR4 levels and thus are more dependent on TLR4 signaling for their survival as compared to other MM cell lines. We also observed that TLR4 activation could protect MM cells from apoptosis induced by ER stressors such as TM. This effect was coupled with ATF4 suppression but not with the downregulation of other UPR branches (e.g. XBP1 and ATF6), indicating that TLR4 signaling may selectively allow plasma cells to exploit the other unaffected UPR pro-survival branches.

TLRs in plasma cells could potentially affect cellular sensitivity to pharmacological substances by inducing resistance to therapeutic agents; thus, we investigated the capacity of TLR4 to abrogate BTZ [a proteasome inhibitor, that is central to multiple myeloma treatment in the clinic^38^] activity in MM cells. As UPR activation increases the proteolytic destruction of misfolded proteins by the proteasome, MM cells rely heavily on proteasomal activity and undergo apoptosis via (among others) terminal UPR when proteasomal proteolysis is disturbed by proteasome inhibitors such as BTZ or other second generation proteasome inhibitors^17,39,40^. We showed that exposure of MM cell lines to LPS, prior to treatment with BTZ, could partially protect cells from BTZ-induced apoptosis; this effect was also evident after TLR4 knockdown in myeloma cells. We further examined the downstream signaling pathway in the UPR system and found that the conferred protection was accompanied with the suppression of CHOP, ATF4, PERK and p-eIF2Α protein expression levels in JJN3 and H929 cells, which were found to be highly dependent on TLR4 signaling as compared to U266 and L363 cells. These findings suggest that the TLR4 ligand may induce the inactivation of signaling pathways which suppress myeloma cells survival leading to development to drug resistance. In support, we found that CD138^+^ plasma cells from BTZ treated patients had increased (*vs*. non treated patients) *TLR4* expression levels; whether the CD138^+^ *TLR4* overexpressing cells survive BTZ therapy and relate to disease relapse remains to be investigated.

In conclusion, our data supports the significance of TLR4 signaling in MM cells biology. Moreover, our results suggest that TLR4 activation may protect MM cells from apoptosis by suppressing the CHOP-ATF4 branch. They thus offer new insights into potential new strategies for MM therapy as it is can be assumed that TLR4 targeting could limit tumor burden and provide an adjuvant therapy to standard treatments.

## Materials and Methods

### CD138^+^ plasma cells and human MM cell lines

Bone marrow from 16 MM patients at diagnosis and after bortezomib (BTZ) treatment, were collected. Mononuclear cells were obtained by gradient centrifugation on Ficoll-hypaque medium (Biochrom, Berlin, Germany). Cells were purified using CD138 microbeads according to manufacturer’s instructions (Miltenyi Biotech, BergischGladbach, Germany).

The MM cell lines U266 (TIB 196) and H929 (CRL-9068) were purchased from ATCC (American Type Culture Collection, Manassas, VA) while the MM cell lines JJN3 and L363 were kindly provided by Pro School of Medicine, fessor C. Mitsiades (Dana-Farber Cancer Institute, Boston, US). MM cell lines were cultured in RPMI 1640 GlutaMAX-I supplemented with 10% heat-inactivated fetal bovine serum (Invitrogen, Paisley, United Kingdom) and 1% penicillin/streptomycin (Biosera, Boussens, France). The ovarian cancer cell line SKOV3 (HTB-77) was kindly provided by Professor Scorilas (Department of Biology, National and Kapodistrian University of Athens, Greece) and cultured in McCoy’s 5a Medium Modified with 10% heat-inactivated fetal bovine serum and 1% penicillin/streptomycin. Cultures were kept at 37 °C in a humidified atmosphere and 5% CO_2_. To deliver a stress response, cells were treated with Tunicamycin [(TM), Sigma-Aldrich] which inhibits N-linked glycosylation and results in the accumulation of ER misfolded proteins. To activate TLR4, cells were treated with LPS (Sigma-Aldrich). The TLR4 inhibitor (TAK-242) was purchased from Invitrogen. Commercially available BTZ was used.

### Flow Cytometry

TLR4 expression before and after LPS stimulation was evaluated by flow cytometry using the CD284 antibody (Biolegend, San Diego, CA) on a CyFlow SL cytometer (SysmexPartec, GörlitzGermany) according to manufacturer’s instructions.

### Cell viability, proliferation and apoptosis assay

The *in vitro* viability assay WST-1 [Cleavage of the tetrazolium salt WST-1 (4-[3-(4-Iodophenyl)-2-(4-nitrophenyl)-2H-5-tetrazolio]-1,3-benzene disulfonate) to formazan] was performed according to manufacturer’s instructions (Clontech, Mountain view, USA). Cells were plated at a density of 10^6^ cells/ml and incubated for 24 hours (h) at 37 °C. Plates were read at 450 nm wavelength on a DAS plate reader (Rome, Italy). Results from WST-1 viability assays are expressed as fraction of treated versus untreated cells. Trypan blue exclusion, as well as Annexin V and propidium iodide (PI) methods were used to determine cell death. The BrdU Cell Proliferation Assay which detects 5-bromo-2′-deoxyuridine (BrdU) incorporated into cellular DNA during cell proliferation using an anti-BrdU antibody, was performed according to manufacturer’s instructions (Cell signaling Technology, Danvers, U.S). Cells were plated at a density of 10^6^ cells/ml and incubated for 24 hours (h) at 37 °C. Plates were read at 450 nm wavelength on a DAS plate reader (Rome, Italy).

### RNA extraction, quantification and amplification

RNA was extracted using the RNA extraction kit (Macherey-Nagel, Düren, Germany) according to manufacturers’ instructions. Quantification was performed using the Qubit spectrophotometer (Invitrogen, Paisley, United Kingdom). cDNA was synthesized from equal RNA amounts using the PrimeScript 1st strand cDNA synthesis kit (Takara, Shiga, Japan).

#### Real-time polymerase chain reaction

cDNA was processed for Quantitative Real Time PCR (Q-RT-PCR) using the SYBR Green Real-Time polymerase chain reaction (Kapa Biosystems, Wilmington, MA) technology according to manufacturers’ instructions. Data were analyzed using the LightCycler 1.5 system (Roche, Basel, Switzerland). Primers used for Q-PCR were designed using the Primer3 software and are as follows (F: Forward, R: Reverse, Sequence: 5′ → 3′) *ATF4*-F: TGAAGGAGTTCGACTTGGATGCC, *ATF4*-R: GAAACCATGCCAGATGACCTTCTG; *CHOP*-F: TGGAAATGAAGAGGAAGAATC AAAAA, *CHOP*-R: CAGCCAAGCCAGAGAAGCA; *TLR4*-F: CTGCAATGGATCAAGGA CCA, *TLR4*-R: TCCCACTCCAGGTAAGTGTT. *β*-*ACTIN* (F: CCCTGGCACCCAGCAC, R: GCCGATCCACACGGAGTAC) was used as Q-PCR normalizer gene.

#### Conventional polymerase chain reaction

Conventional polymerase chain reaction was performed to validate RT-PCR results. For 50 µl reaction, a platinum *Taq* reaction was used containing 4 pmol of primers (Invitrogen) with 1 unit of platinum *Taq* DNA polymerase and 200 µM dNTPs, as per manufacturer’s instructions. The cycling conditions were 95 °C for 2 min followed by 30 cycles of 94 °C for 15 sec; 60 °C for 1 min and 72 °C for 30 sec. PCR products were viewed in 2% ethidium bromide agarose gels.

#### Immunoblotting analysis

Cells were spun at 1000 × g for 5 min and pellets were washed with ice-cold phosphate-buffered saline (PBS). After PBS washes, pellets were re-suspended (100 μl per 1 × 10^6^ cells) RIPA buffer supplemented with a protease/phosphatase inhibitors cocktail (Pierce, Rockford, IL) and left on ice for 30 min. Cell suspension was then spun at 3500 × g for 15 min at 4 °C and the supernatant (protein lysate) was collected and stored at −80 °C. Protein lysates were quantified and equal protein amounts were separated by SDS-PAGE and transferred to polyvinylidenedifluoride (PVDF) membranes. Membranes were blocked with 5% milk and incubated overnight at 4 °C with the primary antibody followed by 1 hour incubation with the secondary antibodies at room temperature. The primary antibodies used were: TLR4 (Novus Biologicals, cat no: NB100-56566), CHOP (Santa Cruz, cat no: sc-575), p-eIF2A (Millipore, cat no: 07-760), eIF2A (Cell Signaling Technology, cat no: 9722), ATF4 (Cell Signaling Technology, cat no: 11815), XBP1 (Santa Cruz, cat no: sc-7160), PERK (Cell Signaling Technology, cat no: 3192), ATF6 (Cell Signaling Technology cat no: 65880), β-Actin (Cell Signaling Technology, cat no: 4967). Used secondary antibodies were anti-rabbit, anti-mouse and anti-goat conjugated to horseradish peroxidase (Cell Signalling Technoloy). Detection was achieved by ECL-Plus (Amersham Biosciences).

#### Plasmid and siRNAtransfection

Transient transfection with pCMV6-TLR4 or a pCMV6 empty vector (OriGene Technologies) was carried out in H929 and U266 MM cell lines cultured in six-well plates by Lipofectamine® 2000 (Invitrogen). For RNAi analyses, JJN3 cells seeded in 6-well plates were transfected by using DharmaFECT Transfection reagent and either the SMARTpool ON-TARGET plus TLR4 siRNA (L-008088-01-0005) or the ON-TARGETplus non-targeting pool (siCtrl) (D-001810-10-05) (GE Healthcare Dharmacon Inc.) according to manufacturer’s instructions.

#### Statistical analysis

Statistical significance was determined using Student’s t test. Data are presented as mean ± standard deviation (SD). Significance at P < 0.05 or P < 0.01 is indicated in graphs by one or two asterisks, respectively. Statistical analysis was performed using Microsoft Excel and GraphPad Prism 5.0 software.

### Ethics approval and consent to participate

An approval from the IRB/Scientific committee of “Alexandra” hospital was obtained for the study and patients gave written informed consent for the collection of samples and their analysis, according to the Declaration of Helsinki.

## Supplementary information

Supplementary information
