# Supplementary material for: Toll-Like Receptor 4 Activation Promotes Multiple Myeloma Cell Growth and Survival Via Suppression of The Endoplasmic Reticulum Stress Factor Chop
Source: Sci Rep. 2019 Mar 1;9:3245. doi: 10.1038/s41598-019-39672-7 (PMC6397208; doi:10.1038/s41598-019-39672-7)
Supplement: Supplementary file 1 — Supplementary information [file 41598_2019_39672_MOESM1_ESM.pdf]

Supplemental information

TOLL-LIKE RECEPTOR 4 ACTIVATION PROMOTES MULTIPLE MYELOMA CELL GROWTH AND SURVIVAL VIA SUPPRESSION OF THE ENDOPLASMIC RETICULUM STRESS FACTOR CHOP

Tina Bagratuni[1], Aimilia D. Sklirou[2], Efstathios Kastritis[1], Christine Ivy Liacos[1], Christina Spilioti[1], Evangelos Eleutherakis-Papaiakovou[1], Nikolaos Kanellias[1], Maria Gavriatopoulou[1], Evangelos Terpos[1], Ioannis P. Trougakos[2], Meletios A. Dimopoulos[1*]

Supplemental Figure S1: Quantification of *TLR4* mRNA expression levels of MM cell lines *vs. β-ACTIN* in agarose gel electrophoresis shown in Fig $1a_1$.

Supplemental Figure S2: Quantification of TLR4 protein expression levels *vs.* β-*ACTIN* in immunoblots shown in Fig $1a_2$.

Supplemental Figure S3: Quantification of *TLR4* mRNA expression levels of MM CD138$^+$ selected cells *vs. β-ACTIN* in agarose gel electrophoresis shown in Fig b.

Supplemental Figure S4: Proliferation of MM cells exposed to increasing doses of LPS for (a) 24, (b) 48 and (c) 72h using the BrdU proliferation assay.

Supplemental Figure S5: Quantification of TLR4 protein expression levels *vs.* β-*ACTIN* in immunoblots shown in Fig 2b.

Supplemental Figure S6: Quantification of TLR4 protein expression levels *vs.* β-ACTIN in immunoblots shown in Figs $3a_2$ (TLR4 siRNA) (a) and $3d_2$ (TLR4 overexpression) (b).

Supplemental Figure S7: Quantification of the indicated proteins expression levels *vs.* β-ACTIN in immunoblots shown in Figs 5b (TLR4 siRNA) (a) and 5D (TLR4 overexpression) (b).

Supplemental Figure S8: Quantification of ATF4-CHOP branch protein markers of MM cells *vs. β-ACTIN* in immunoblots shown in Fig 6B.

Supplemental Figure S9: Protein expression of integrated stress response selected markers in JJN3, H929, L363 and U266 cells before and after LPS (1 μg/ml) treatment for 24 h. Probing with β-ACTIN was used as total protein loading reference.

Supplemental Figure S10: Schematic representation demonstrating the possible interaction of TLR4 activation with integrated stress responses.

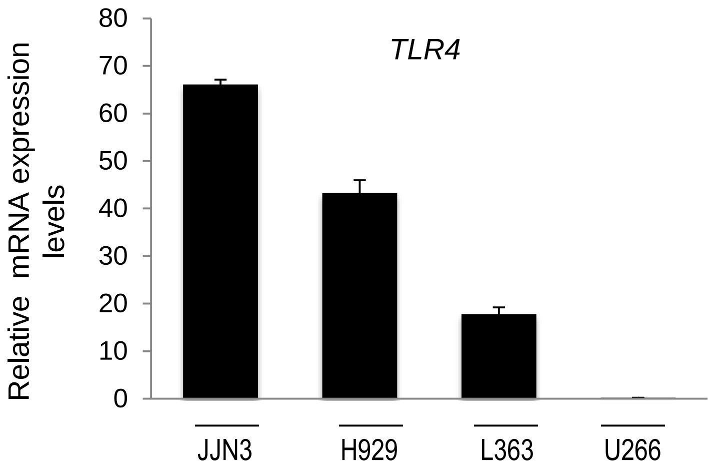

Bagratuni et al. Suppl. Fig. S1

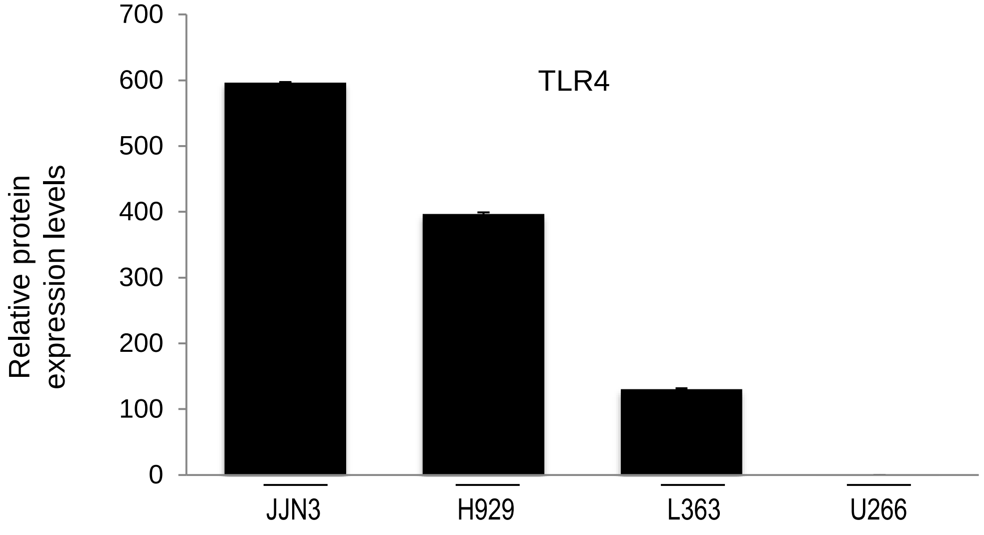

Bagratuni et al. Suppl. Fig. S2

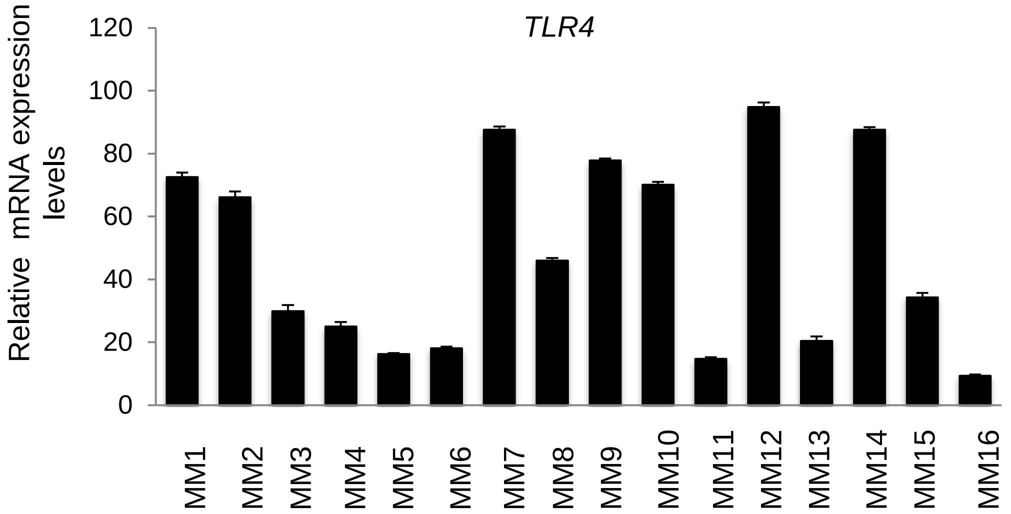

Bagratuni et al. Suppl. Fig. S3

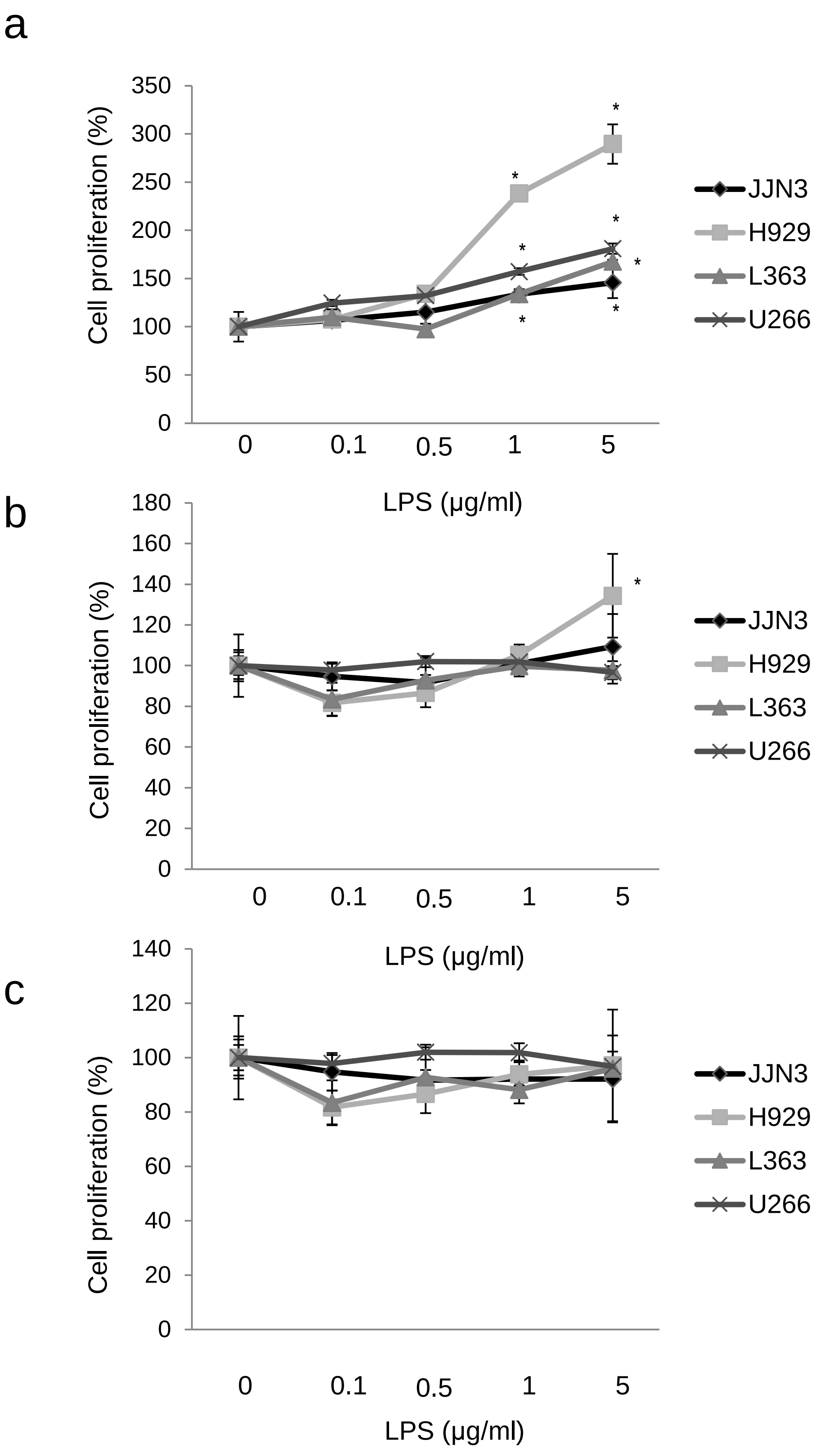

Bagratuni et al. Suppl. Fig. S4

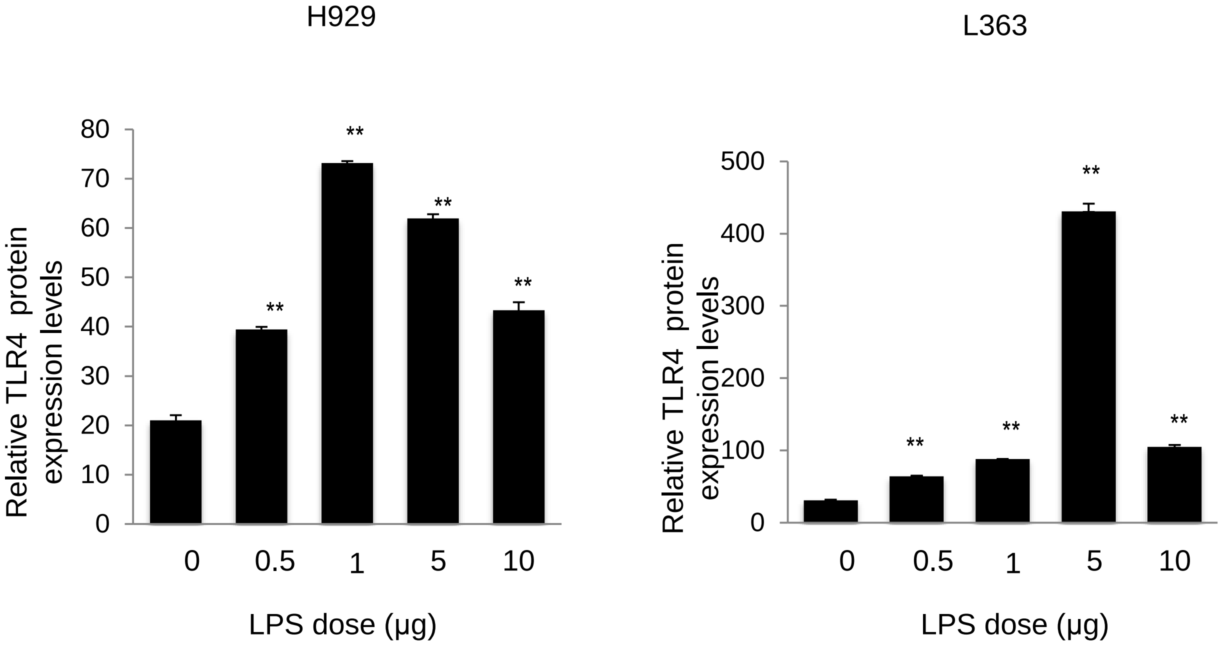

Bagratuni et al. Suppl. Fig. S5

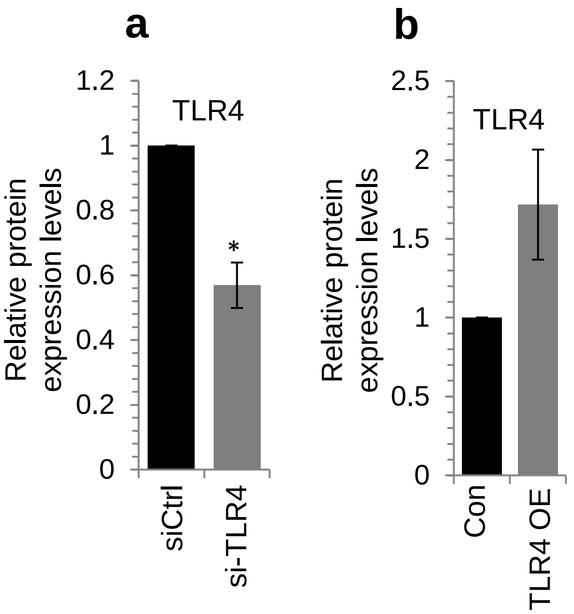

Bagratuni et al. Suppl. Fig. S6

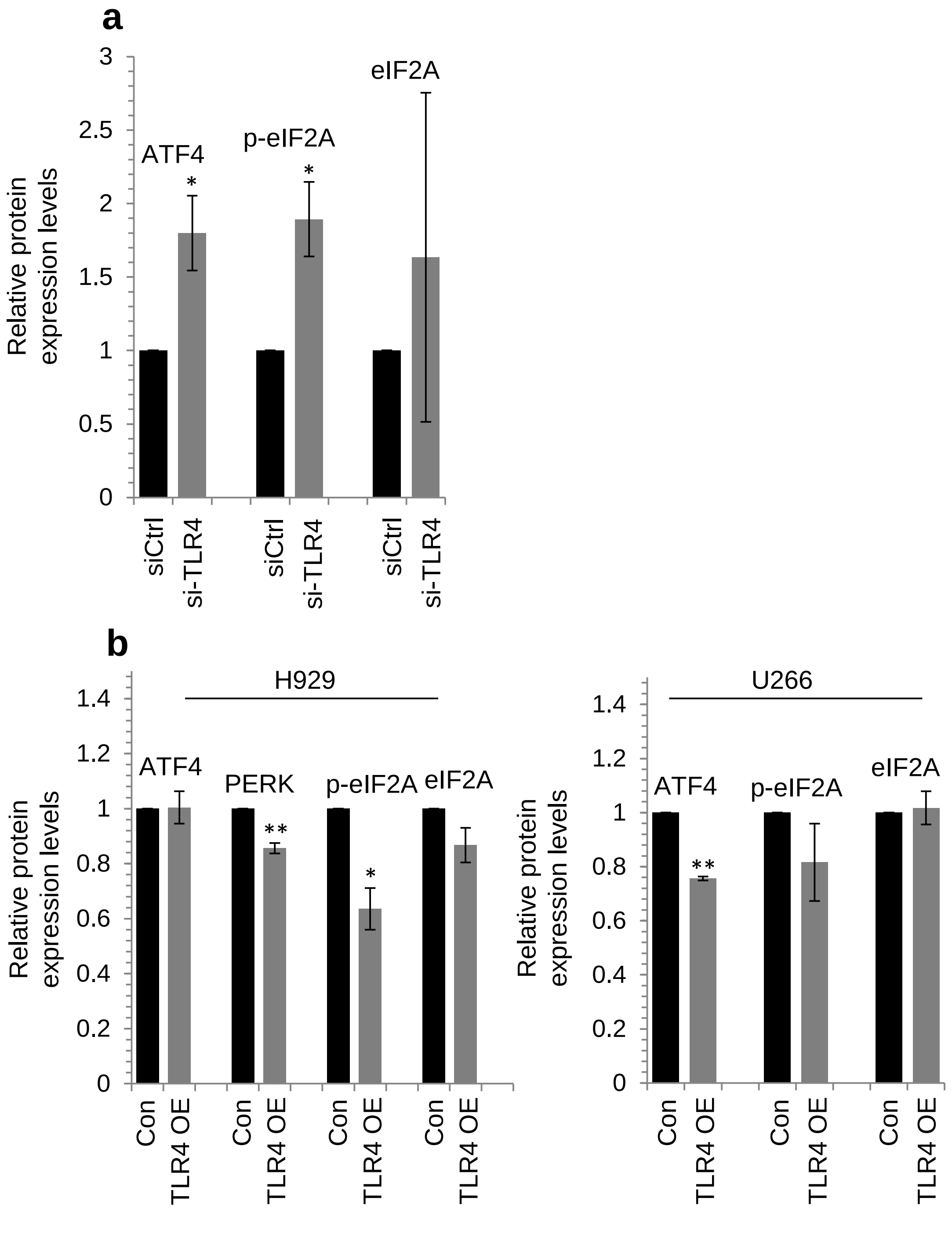

Bagratuni et al. Suppl. Fig. S7

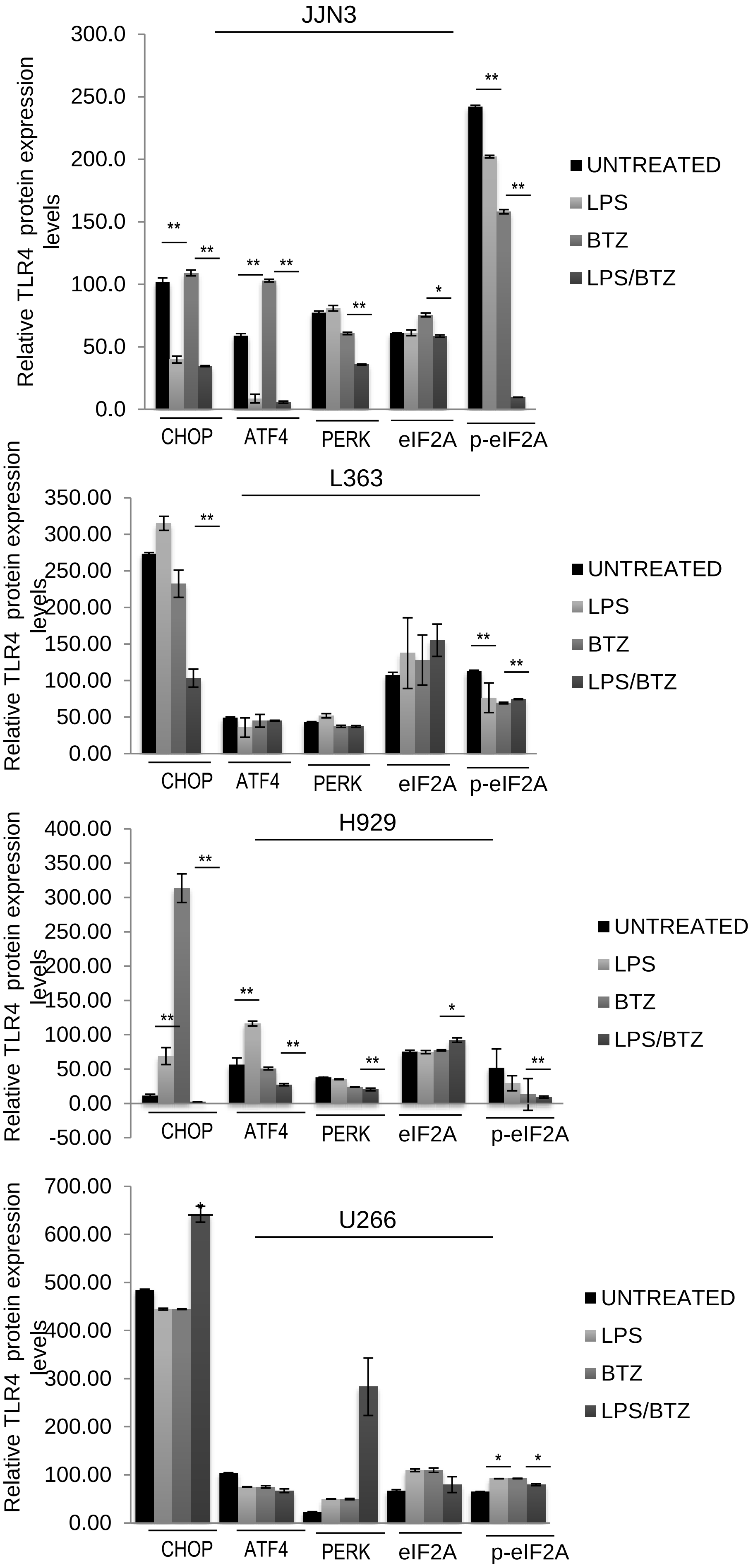

Bagratuni et al. Suppl. Fig. S8

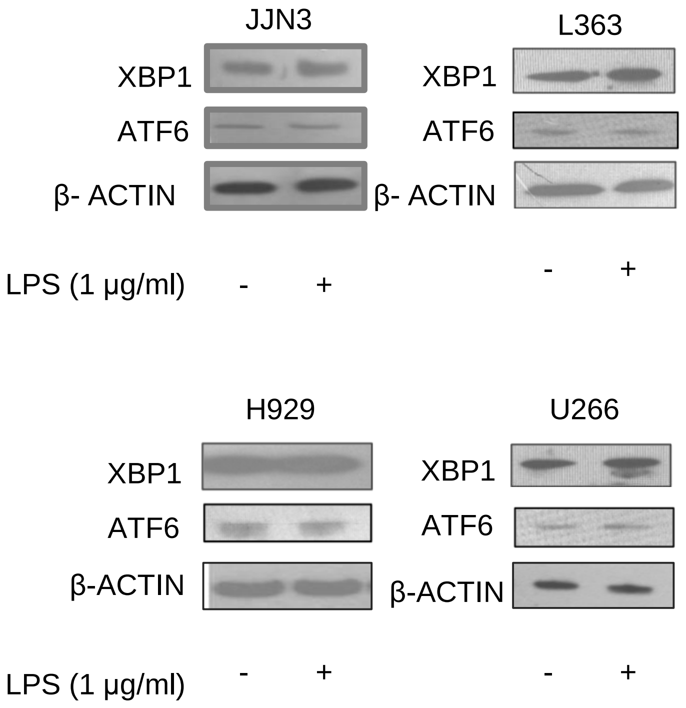

Bagratuni et al. Suppl. Figure S9

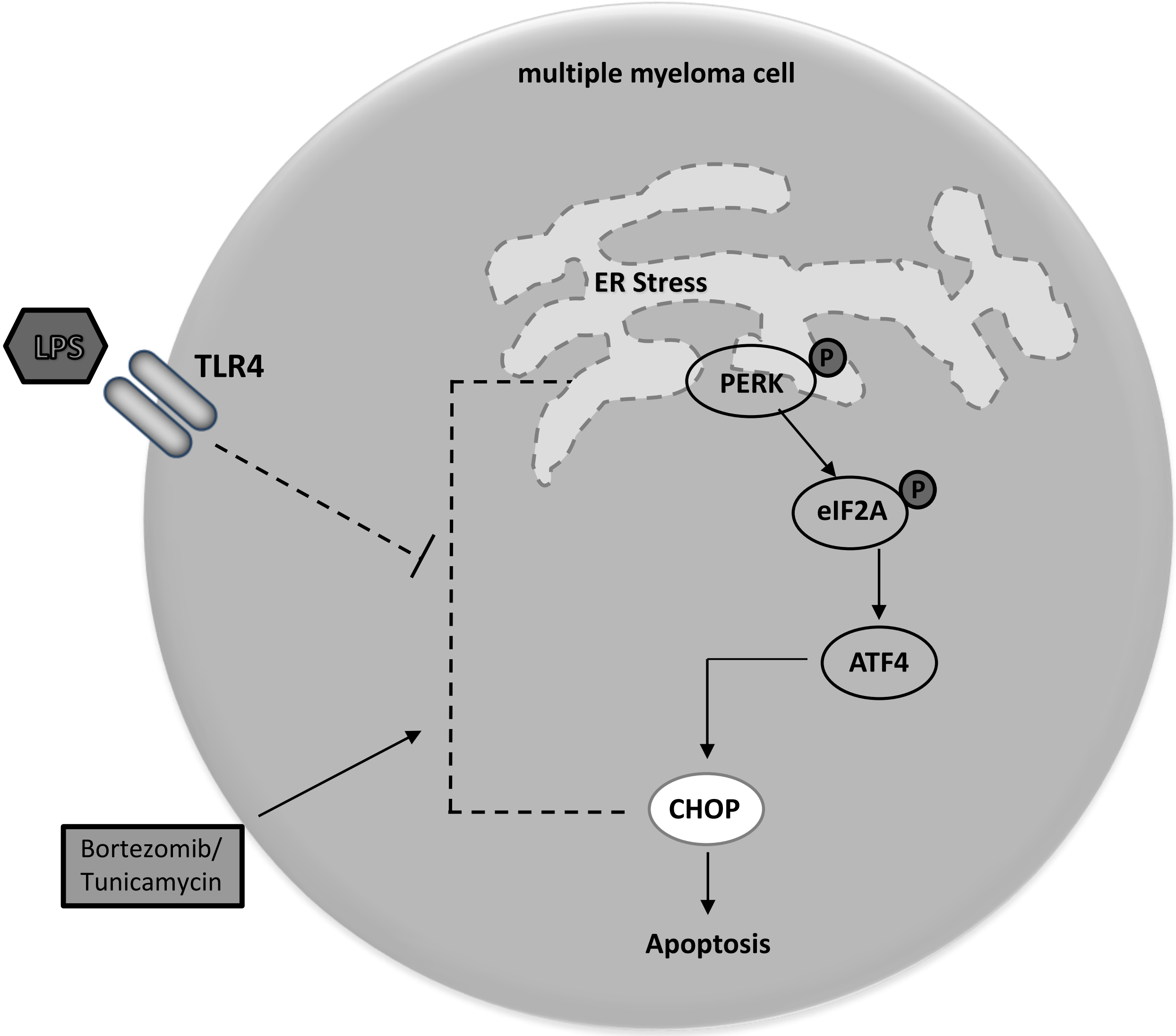

Bagratuni et al. Suppl. Fig. 10

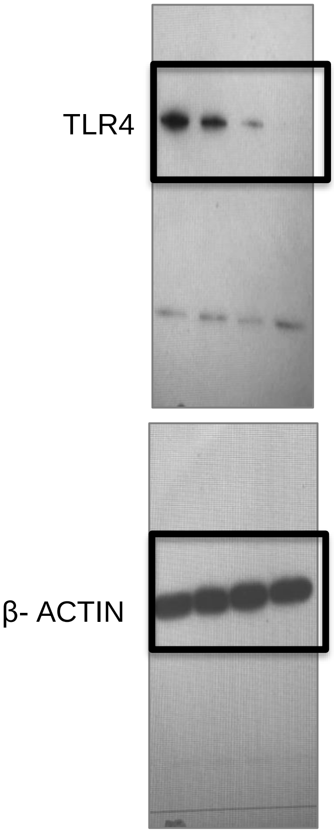

Figure $1a_1$

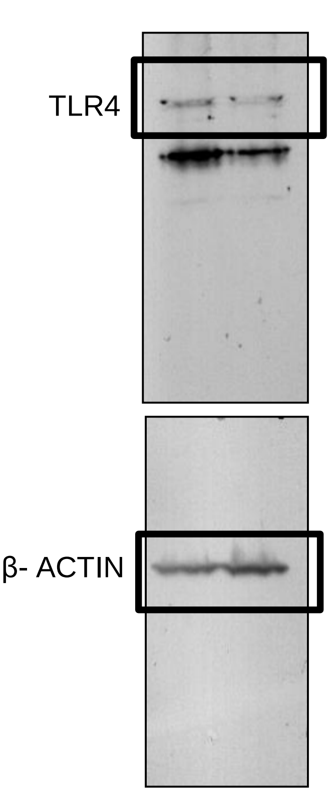

Figure $3a_2$

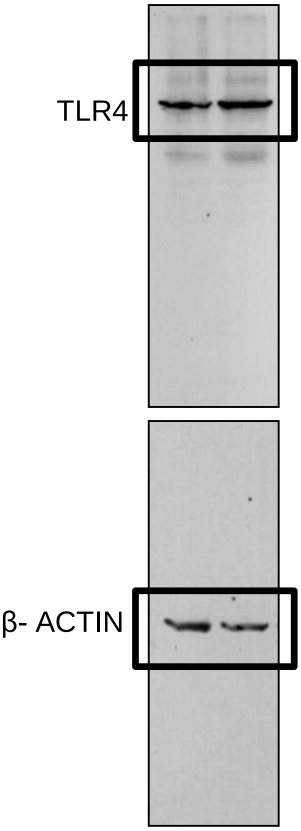

Figure $3d_2$

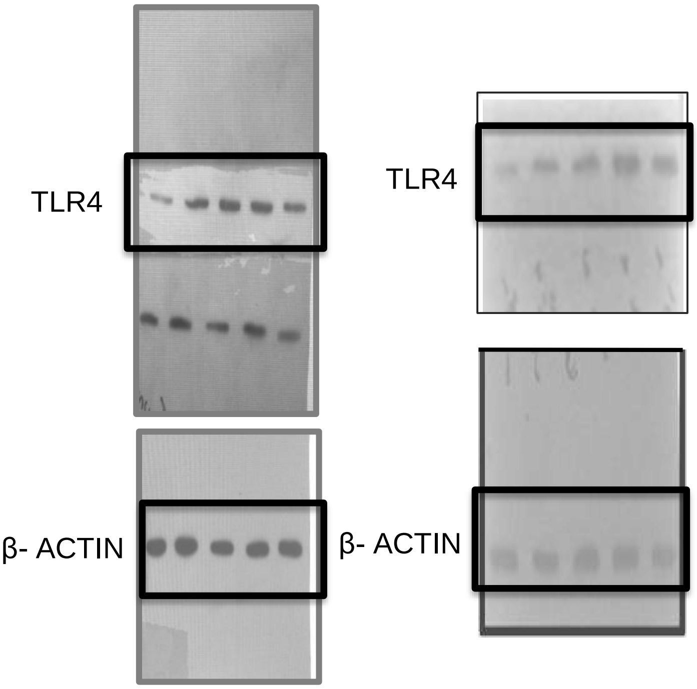

Figure 1b

Bagratuni *et .al.*Supplemental data S11

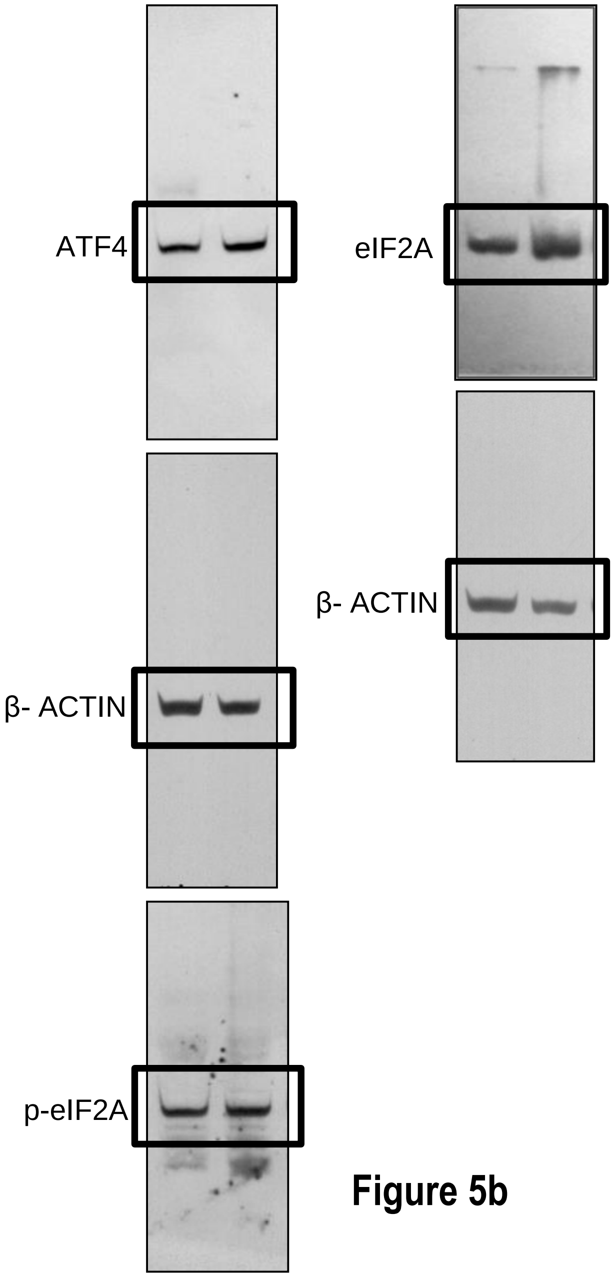

Figure 5b

Bagratuni *et .al.*Supplemental data S11

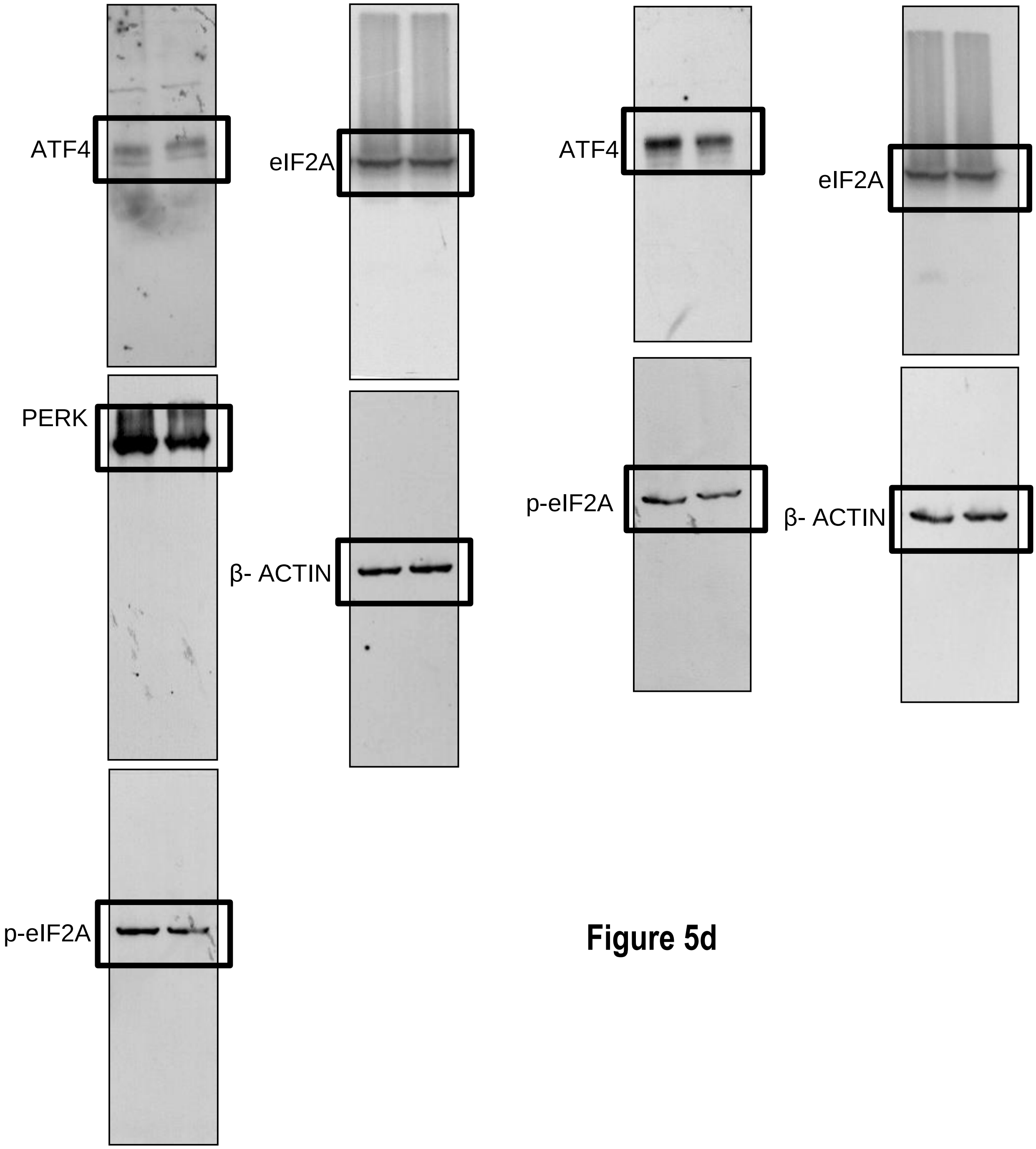

Figure 5d

Bagratuni *et .al.*Supplemental data S11

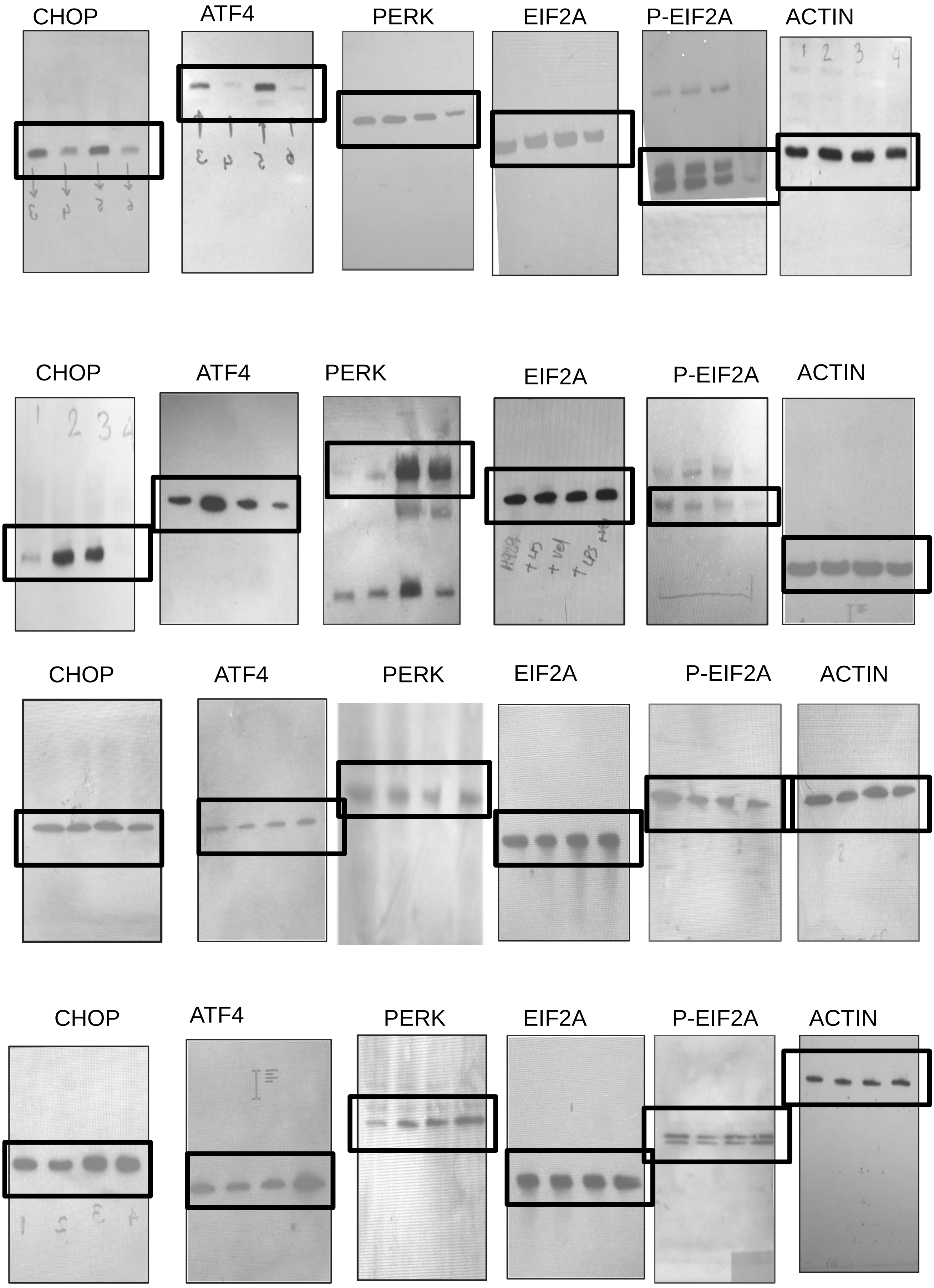

Figure 6b

Bagratuni *et .al.*Supplemental data S11
